# Food Related Quality of Life in Children with Chronic Gastrointestinal Disorders: Comparative Study Between Inflammatory Bowel Disease and Celiac Disease—Reports from a Romanian Center

**DOI:** 10.3390/nu17010051

**Published:** 2024-12-27

**Authors:** Roxana Elena Matran, Andra-Mihaela Diaconu, Andreea Maria Iordache, Daniela Pacurar, Cristina Adriana Becheanu

**Affiliations:** 1 Department of Paediatrics, “Carol Davila” University of Medicine and Pharmacy, 050474 Bucharest, Romania; elena.smadeanu@umfcd.ro (R.E.M.); danapacurar@yahoo.com (D.P.); cbecheanu@yahoo.com (C.A.B.); 2 “Grigore Alexandrescu” Emergency Hospital for Children, 011743 Bucharest, Romania; andreeamariavlad@yahoo.com

**Keywords:** quality of life, inflammatory bowel disease, Crohn’s disease, ulcerative colitis, celiac disease, nutrition, dietary interventions, FR-QoL

## Abstract

**Background/Objectives:** Chronic gastrointestinal disorders often involve nutritional management strategies. On the one hand, inflammatory bowel disease (IBD) is a condition in which most of the patients experience frequent diet manipulation in order to obtain long term remission. On the other hand, for celiac disease (CelD), diet is the only known treatment strategy so far, requiring a life-long gluten-free diet. We aimed to evaluate the comparative food-related quality-of-life (FR-QoL) in light of these dietary interventions between these two conditions. **Methods:** This is a cross-sectional study, involving children aged 7–18 years diagnosed with IBD and CelD. Assessment of this aspect was performed using the self-reported FR-QoL 29 questionnaire. For CelD, the questionnaires were modified with “CelD” instead of “IBD”. **Results:** Fifty-one patients were included, 17 in each subgroup (Crohn’s disease (CD), ulcerative colitis (UC), and Celd). FR-QoL scores were negatively correlated with age at inclusion (Spearman’s ρ = −0.284, *p* = 0.04) and also with age at diagnosis (Spearman’s ρ = −0.291, *p* = 0.038). The scores were significantly lower in the CD group (64.1 ± 13.4) compared with CelD patients (78.6 ± 20.3), *p* = 0.036 and UC, *p* = 0.294. For the IBD group, the scores were not influenced by disease activity. Furthermore, we identified a negative significant correlation between anthropometric indices and FR-QoL scores. **Conclusions:** The burden of dietary intervention is highest for the CD patients, regardless of their disease activity when compared with UC and CelD patients, most probably because of the unpredictable course and fast response to dietary changes. Although it requires incessantly vigilant eating behavior, CelD has apparently become more “manageable” in recent years.

## 1. Introduction

Celiac disease (CelD) and inflammatory bowel diseases (IBD) are gastrointestinal disorders, characterized by abnormal immune responses, resulting in disrupted immune pathways [1]. 

Pediatric inflammatory bowel disease (pIBD) is the most common chronic gastrointestinal disorder under the care of pediatric gastroenterologists, with a marked increase in the incidence and prevalence globally in recent decades [2,3]. The main spectrum of pIBD includes three main entities: Crohn’s disease (CD), ulcerative colitis (UC) and inflammatory bowel disease—unclassified (IBD-U)—according to the European Society of Pediatric Gastroenterology, Hepatology and Nutrition (ESPGHAN) Porto criteria [4]. Based on the current literature, diet plays a crucial role in IBD pathogeny and hence represents an encouraging target for therapeutic strategies, with an increasing interest in research in this domain being observed recently. Recent reports by Gerasimidis et al. suggest that with the exception of exclusive enteral nutrition (EEN) indicated for active CD, there is currently insufficient evidence to formulate other specific dietary recommendations for the management of pIBD [5]. However, patients with IBD are frequently exposed to diet manipulation during the disease course, both in UC and CD whether as a specific intervention or as adjunct therapy, in order to enhance the response to standard medical regimens or in an effort to manage associated functional abdominal symptoms [6].

Ishige et al. report that at diagnosis, 3 to 10% of the children diagnosed with UC and 15 to 40% with CD are associated with growth failure [7]. They further note that the risk is highest among patients with pre-pubertal onset CD, potentially resulting in a final adult height up to 8 cm lower than controls [2]. Moreover, malnutrition may be the consequence of the combination of disease activity and dietary restrictions [8]. 

Food and eating habits serve multiple purposes beyond their fundamental physiological functions. While essential for ideal growth and development and in our long-term health, food is involved also in our social life and is also capable of influencing our emotions and our day-to-day living. These aspects collectively define food-related quality of life (FR-QoL). A diagnosis like IBD may disrupt these psychosocial components of eating, leading to an avoidance of different social, cultural or religious events involving food and moreover, shifting the perception, with food and often shopping for groceries or preparing a meal becoming sources of concern rather than enjoyment [9].

To measure the FR-QoL in IBD, a specific questionnaire FRQoL-29 was developed and validated in adult patients with IBD, which was used subsequently in numerous studies. An agreed conclusion is that patients with IBD experience decreased FR-QoL compared with healthy controls, or other chronic diseases like asthma or when compared to functional disorders of the gastrointestinal tract [10,11]. 

CelD is triggered by gluten exposure in a genetically susceptible population, HLA-DQ2 and HLA-DQ8 being the genotypes involved. The diagnosis is established characteristically within the first two years of life, after complementary feeding initiation, with another peak-onset later in life, in the second decade [12,13]. A diagnosis of CelD is confirmed using European Society of Gastroenterology, Hepatology and Nutrition’s (ESPGHAN) recommendations and is based on a combination of clinical, serologic and histopathological changes [14]. Growth impairment is a well encountered manifestation in CelD. In a recent study by Almahmoud et al., failure to thrive was the most prevalent symptom at diagnosis, reported in 31% of the cases [15]. It is well known that a gluten-free diet (GFD) is, at this moment, the only treatment strategy for CelD patients, involving major dietary limitations [16]. Dietary adherence in children ranges widely from 23 to 98%, regardless of the technique used to assess compliance [17]. Existing research suggests that GFD is associated with potential risk factors like trace element deficit, excessive carbohydrate and lipid intake and fatty liver disease [18]. 

For CelD, there is no formulated unidimensional instrument for specifically assessing the FR-QoL. HR-QoL was proposed to be evaluated with the specific celiac disease Dutch questionnaire (CDDUX), formulated in 2008 by van Doorn et al., in a study population that included patients with CelD and a control group consisting of patients with asthma, rheumatoid arthritis and diabetes. The questionnaire includes 12 items divided into three different scales, a “communication” scale, a “having CelD” scale and a “diet” scale [19].

As stated above, although nutritional interventions are the only therapeutic intervention for CelD, this requires lifelong adherence to specific cautious and also challenging eating behaviors. In comparison, most pIBD patients, including UC are, at some point in their evolution, subjected to diet interventions either indicated by the treating physician or self-initiated. 

The existing literature indicates that FR-QoL is impaired in children with CD when compared to the healthy population. However, studies comparing FR-QoL between the two main types of IBD, or between IBD and other organic gastrointestinal disorders to our knowledge, are lacking. 

Our aim is to evaluate the FR-QoL and compare its impact across the two main entities of IBD, UC and CD in which most children undergo dietary challenges disregarding the disease form, as well as CelD, a condition where dietary management is the cornerstone. Furthermore, as second objectives, we evaluated the possible factors that may influence FR-QoL, in order to intervene in a timely manner to prevent further deterioration of this particular aspect of the global management of these chronic disorders.

## 2. Materials and Methods

We conducted a cross-sectional study including 51 children diagnosed with IBD and CelD being followed in the “Grigore Alexandrescu” Emergency Hospital for Children in Bucharest, Romania, performed between April 2024 and November 2024. Study population was further divided in three subgroups, 17 children with CD, 17 children diagnosed with UC and 17 children with CelD. Children were included in the study if they were diagnosed with IBD at least 3 months prior to inclusion according to ESPGHAN guidelines [20], while patients included with CelD had to be diagnosed at least 6 months before enrollment, according to ESPGHAN guidelines [14]; all children were included in the study during their routine disease assessment, if they were 7 years or older. The exclusion criteria were the following: exclusive enteral nutrition (EEN), use of parenteral nutrition (total or partial) or surgery in the last 3 months for IBD subgroup and incomplete ESPGHAN criteria for diagnosis of CelD in this subgroup of patients. The number of patients in each group was determined by the final number of CD patients enrolled. From 87 IBD patients followed in our clinic, 22 were diagnosed with CD; 2 patients were under EEN, 2 patients were under 7 years of age and 1 patient refused to be enrolled. The remaining 17 patients were enrolled in the final CD subgroup. Seventeen patients were enrolled for the UC and CelD subgroups patients according to the inclusion and exclusion criteria described above. Consent from their legal guardian was obtained for all the children. 

Participants were provided with the translated Romanian version of the questionnaire at the time of their evaluation and were asked to complete it at home, within a week at most. For the CelD patients, questionnaires were modified with “CelD” instead of “IBD”. The completed questionnaires were electronically returned for ease of collaboration. All children completed the survey by themselves except for one 8-year-old patient in the IBD subgroup and two children, an 8-year-old boy and a 7-year-old girl with CelD who required assistance from their parents. 

The self-reported FR-QoL-29 questionnaire comprises 29 items with a 5-point Likert response scale, ranging from 1 (definitely agree) to 5 (definitely disagree) evaluating different aspects of eating and drinking, in the last two weeks. Results range from a maximum score of 145 points, indicating unaffected FR-QoL, to 45 points denoting a substantial negative impact [10]. Four questions (8, 9, 24, 25) are positive expressions so, when calculated, the score was reversed in order to associate the highest scores with a positive response and, respectively, the lowest ones with a negative one for the accurate appreciation of internal consistency within the questionnaire.

Demographic and clinical data were collected. The disease extension and behavior for IBD patients were categorized according to EPSGHAN’s Paris classification [20]. Additionally, history of surgery and medical treatment regimens were noted. For the CelD subgroup, adherence to GFD was assessed using the tissue transglutaminase testing IgA or IgG, in the case of IgA specific deficit and patients’ self-reports. 

Anthropometric (weight, height) data were collected and the nutritional status was appreciated using the conversion of the body mass index (BMI, kg/m^2^) into standardized Z-scores and classified according to the Centers for Disease Control and Prevention (CDC) references for children 2–20 years, as follows: BMI < 5th percentile—“underweight”, BMI ≥ 5th to <85th percentile—“healthy weight”, BMI ≥ 85th percentile to <95th percentile—“overweight”, BMI ≥ 95th percentile to <120% of the 95th percentile and <35 kg/m^2^—“class I obesity”, BMI ≥ 120% to <140% of the 95th percentile or ≥35 kg/m^2^ (whichever is lower)—“class II obesity” and BMI ≥ 140% of the 95th percentile or ≥ 40kg/m^2^ (whichever is lower)—“class III obesity” for a uniform assessment [21]. For IBD patients, disease activity was evaluated using the Physician Global Assessment (PGA) and the specific activity indexes like Pediatric Ulcerative Colitis Index (PUCAI) for UC and weighted Pediatric Crohn’s Disease Activity Index (wPCDAI) for CD. According to PGA, the overall disease activity was categorized into quiescent = 0, mild = 1, moderate = 2 and severe = 3 [22].

The wPCDAI is measured on a 0 to 125 scale, <12.5 points indicating remission, 12–40 points indicating mild activity, >40–57.5 points indicating moderate disease and scores higher than 57.5 up to the maximum of 125 being indicators of severe disease [23]. The PUCAI score classifies UC as remission < 10 points, mild activity 10 to 34 points, moderate activity 35 to 64 points and scores > 65 points (up to 85) indicating severe colitis [24]. Treatment was categorized into Crohn’s disease exclusion diet (CDED) in association with partial enteral nutrition (PEN), 5-aminosalicilates (5-ASA), immunomodulator (IMM, azathioprine), infliximab (IFX) and adalimumab (ADA) and strict GFD for CelD patients. Patients receiving more than one intervention were included in each corresponding category. 

The study was approved by the Ethics Committee of “Grigore Alexandrescu” Emergency Children’s Hospital (# 43/31 October 2024) and conducted in accordance with the Declaration of Helsinki. Patient’s confidentiality and privacy were maintained. 

### Statistical Analysis

Data were analyzed using SPSS v26 (Chicago, IL, USA). Descriptive statistics were computed for both continuous and categorical variables. Continuous variables were evaluated for normality using the Kolmogorov–Smirnov test. For continuous variables, the mean/median with standard deviations (SD) or interquartile range (IQR) as appropriate were used, regarding their distribution. For categorical variables both the Chi-square test and Fisher’s Exact Test were performed. Fisher’s Exact Test was preferred in cases where sample sizes were small or where expected frequencies in contingency table cells were below 5, ensuring robustness and accuracy of the *p*-values. For the not normally distributed data, the Mann–Whitney U Test (a non-parametric test) was applied to compare medians between groups. To compare means of continuous variables between more than two groups, one-way Anova was used for the normally distributed data and the Kruskal–Wallis one-way for the non-normally distributed data. Independent-T-test was used to compare means of normally distributed variables. Pearson and Spearman’s nonparametric correlation coefficient test were employed to assess relationships between different variables according to the type of data distribution. Post hoc analysis was performed for ANOVA and Kruskal–Wallis, when significant differences were found between groups. A *p* value < 0.05 was considered as the threshold for statistical significance. Internal consistency of the instrument was evaluated using Cronbach alpha. The accepted 0.7 threshold indicating a good internal consistency was used.

## 3. Results

A total of 51 children were enrolled; the median age [IQR] at inclusion was 15 [12–17] years; 17 were diagnosed with CD, 17 with UC, respectively, and 17 patients were diagnosed with CelD, with 41.2% (n = 21) boys in the entire study population. The median [IQR] age at diagnosis was 11 [4.3–14] years, significantly lower for CelD patients. Ileocolonic extension was the most frequent disease involvement for CD, with inflammatory (B1) behavior. For UC, pancolonic involvement was the most prevalent. The duration of evolution until enrollment was significantly longer in the CelD subgroup. A strict GFD was for the majority of CelD patients, 94.1% (n = 16). Most of the patients were in the “normal weight” category. The patients’ characteristics are described in Table 1. 

To evaluate the internal consistency of the questionnaire items, Cronbach’s alpha was calculated for the 29 items’ responses. The analysis yielded a Cronbach’s alpha of 0.910, indicating a high level of internal consistency. This suggests that the items are highly correlated and reliably measure the same underlying construct. 

**Figure 1:**
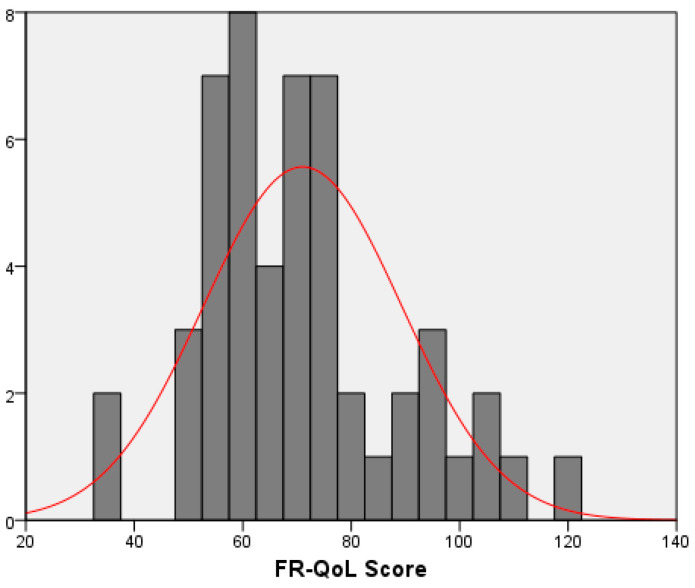
Distribution of the FR-QoL scores in the population study. FR-QoL, food-related quality of life.

Overall, the entire study population associated a relatively low mean (SD) FR-QoL score of 71.03 ± 18.3 (Figure 1). Scores were similar between genders, 71.2 ± 18.6 for girls and 70.9 ± 18.3 for boys, *p* = 0.953.

FR-QoL scores were negatively correlated with age at inclusion (Spearman’s ρ = −0.284, *p* = 0.04) and with age at diagnosis (Spearman’s ρ = −0.291, *p* = 0.038). No significant correlation was observed between the scores and duration of evolution, Spearman’s ρ = −0.170, *p* = 0.234. 

Most patients, both with CD and UC, were in remission (76.5%, n = 26), according to specific activity scores. Median PUCAI was 0 [0–40] while median wPCDAI was 5 [0–7.5]. No differences were observed in the FR-QoL scores between the two subgroups (66.2 ± 17.8 for the remission group and 77.25 ± 15.2 for the active disease group, *p* = 0.124). History of surgery did not influence the scores (61.83 ± 7 for the positive history group vs. 70.32 ± 18.9 for the negative group, *p* = 0.291). Similar findings were observed when analyzing the extension or the behavior of the disease (Table 2). 

**Figure 2:**
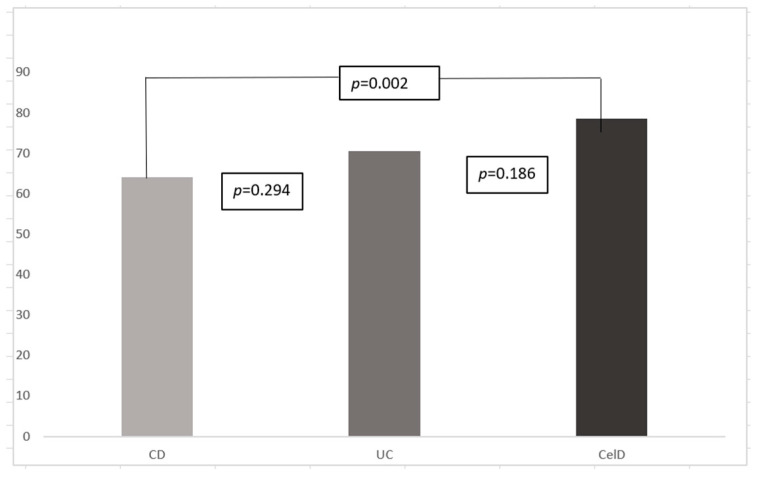
Fr-QoL scores according to disease type. CD, Crohn’s disease; UC, ulcerative colitis; CelD, celiac disease.

According to disease type, scores were significantly lower for the IBD subgroup, 67.3 ± 16.1 for IBD vs. 78.6 ± 20.3 for the CelD patients, *p* = 0.036. At a further post hoc analysis, between all three diseases subtypes, only CD scores (64.1 ± 13.4) differed significantly from the CelD results, *p* = 0.02, with no statistical difference between IBD subgroups (70.5 ± 18.2 for UC patients), *p* = 0.294 nor between UC and CelD patients, *p* = 0.186 (Figure 2). 

The majority (80.4%, n = 41) of patients included in the study were categorized as “healthy weight”. Mean FR-QoL scores were similar between the “normal/overweight” and “underweight” groups, 70.3 ± 17.9 vs. 74.1 ± 20.3, *p* = 0.560.

Interestingly, scores were negatively correlated with weight, Pearson’s r = −0.347, *p* = 0.013 and with height, Spearman’s ρ = −0.364, *p* = 0.009.

## 4. Discussion

To our knowledge, this is the first study to address the evaluation of FR-QoL between two chronic gastrointestinal disorders in pediatric pathology, particularly in Romania. Although not designated for CelD, upon completion of the FR-QoL by CelD patients, some comparison can be made because of similar food-related concerns, in the absence of a specific construct that addresses this issue entirely. 

Several authors highlighted the aspect of living with CelD in recent papers. Besides the periodic medical assessments, potential complications and lifelong monitoring, CelD also implies a high level of dietary caution. Anxiety about cross-contamination of certain foods and the possible occurrence of digestive and also extraintestinal symptoms associated with ingesting gluten-contaminated food, represent a possibility for developing eating disorders. Constant checking of ingredient lists, food menus and the avoidance of public dining situations can limit socializing and exposure to potential beneficial experiences while increase the feeling of discrimination [25,26].

Because of lifelong, strict interventions in an area which is an essential part of day-to-day living, in terms of quality of life (QoL) the GFD burden becomes obvious and besides specific evaluation, patients with CelD need to be assessed from this point of view also [27].

The family’s perception of CelD’s impact is different. Mothers report greater lifestyle changes and the burden of being challenged with managing gluten-free diets while fathers experience guilt for their genetic contribution [17].

In a recent study that analyzed the adjustments that children and adolescents with CelD make in order to take part in various activities including food, the author found that almost all patients “always” adhere to the diet and their actions involve cognitive strategies that extend well beyond simply refraining from gluten-containing food [28].

The particular aspects of adolescence as a time of emotional transition, which coincides with the peak incidence of IBD, makes the diagnosis of a chronic illness in this time-period have a significant impact from a psychosocial point of view, not only on patients but also on the family [29,30]. 

From the patient’s perspective, living with IBD implies lifestyle adjustments and behavior adaptation regimens. Daily medication, periodic medical assessments and last, but not least, dietary interventions which may vary roughly depending on the disease evolution and on the health-care provider, are common aspects among IBD patients [31].

Patients often turn to the internet for dietary guidance when comprehensive advice from the treating physician on appropriate diets is lacking, which may prove to be a source of misinformation [32]. Diet becomes a major concern for patients with IBD, either because of the specific recommendations and restrictions or because they believe that this is safer than the medical therapies, with 60% of them report controlling their symptoms and extending the periods of remission through manipulation of diet, mainly by avoiding foods and drinks observed to trigger intestinal symptoms; an adaptation mechanism defined as perceptive eating [31,33,34]. Finding what food they can and cannot tolerate is a process of “trial and error”, as suggested by de Vries et al. [33]. In order to limit their intestinal symptoms, guided by past experiences or driven by the anticipated fear of harmful effects, patients may even develop eating behaviors like avoidant/restrictive food intake disorders (ARFID) [35]. 

Following diagnosis, approximately half of the patients with IBD modify their dietary habits, implementing varying degrees of food restrictions, ref. [36] with most adults avoiding spicy food and gluten, while identifying rice as the primary dietary component believed to prevent flare-ups and alleviate symptoms [37]. Children with IBD report an avoidance behavior towards foods in approximately half of the cases. According to Diederen et al., common avoided foods were as follows, in descending order: spicy food, fat-dense products, dairy, cereal-based products, onions/leeks, bell peppers, refined sweets, meat and sodas; while their diet is rich in vegetables, monounsaturated, and poly-unsaturated fatty acids [38]. In our study, we observed a tendency towards lower scores, irrespective of the disease. As reported recently by Subramanian et al., both adults and children with autoimmune gastrointestinal diseases (CD, UC, CelD) are at risk of developing eating disorders, the risk in children being more than twice higher comparative with children with non-immune digestive disorders [39]. These reports may underestimate the true prevalence of the wide range of possible eating disorders because the study takes into consideration only disorders diagnosed and classified according to ICD-9 codification. 

Our results indicate that FR-QoL scores were increasing as the age at inclusion and age at diagnosis were lower. We speculate this may indicate that at younger ages, the family may take over the burden of the diet, especially in this time-frame when social activities including food are not yet intensely and independently explored. Parents of IBD patients regard diet as a challenging situation after IBD diagnosis; they are dissatisfied by the lack of personalized dietary interventions and by the inconsistences of provided nutritional advice [40]. Furthermore, a younger age at diagnosis allows patients to become accustomed to a specific diet intervention that becomes a default way of living. This is contrary to what Brown et al. observed in their study that included pediatric CD patients and their healthy siblings, that the FR-QoL scores increased as the age was older, suggesting the development of coping techniques in relation to their eating behavior over time [41]. 

Our results show significantly lower FR-QoL in patients with CD when compared with the UC group and CelD. These are in accordance with previous reports of the reduced impact of diet in patients with UC, compared to CD [36]. Previous studies in adults, indicate that once adherence to GFD is achieved, HR-QoL shows improvement at the same or even higher level than the general population [18]. These last observations could be attributed to the increased accessibility of gluten-free products. The gluten-free product market is worth almost USD seven billion and is expected to grow up to 14 billion in the next 10 years [42]. Also, better tasting versions of such products have been attained. Together, these achievements have transformed the GFD into an easier-to-bear dietary intervention [43]. Furthermore, despite the significant dietary modifications and adjustments required in food-related situations, children with CelD report medium to high HR-QoL [44]. 

Factors that influence FR-QoL were analyzed in a recent systematic review by Zhu et al. that comprised five studies, The authors reported a positive correlation in children with CD between FR-QoL and age, weight, height, and BMI. They also observed that patients in remission presented with higher FR-QoL scores than patients with active disease [45]. Furthermore, Jiang et al. reported in a study comprising adults with IBD that for UC the activity and endoscopy scores indicating active disease correlated with lower FR-QoL scores [46].

In the current study, we did not observe any correlation between disease activity and FR-QoL scores, most probably because most patients were in clinical remission. Further analysis including endoscopic scores or mucosal inflammation markers, like fecal calprotectin, may be needed in order to precisely evaluate the FR-QoL in relation to disease activity in larger populational samples. 

Counterintuitive at first sight, we report negative correlations between FR-QoL scores in CD patients and anthropometric indices like weight, height and BMI, but not with the Z-score for BMI, contrary to the findings of Brown et al., which reported positive correlations [41]. One possible explanation for our observations may be the fact that most patients in our study were within normal weight range and we did not identify any correlation with disease activity, which may be a potential confounder. 

Limited research exists on FR-QoL in pediatric patients, with most comparisons drawn from adult populations. However, various observations provide more accurate insights into the diversity of factors that may influence FR-QoL. 

Although valuable to the not so well-known general picture of FR-QoL in children with chronic gastrointestinal diseases, our study has some limitations. First of all, we are facing a relatively small sample size of the study population in each subgroup. The self-report nature of the assessment may entail validity issues, as exaggerating or underreporting of symptoms, dependent on the patient’s disposition at the time of completing the questionnaire, or as described in the medical literature because of a behavior similar to the “Hawthorne effect” [47], which may be a source of biased responses caused by changes in children’s attitude in the context of a medical evaluation. A longitudinal evaluation through repeated questionnaires at different times would be more accurate in evaluating the FR-QoL. Also, we did not take into account the patients’ intellectual limitations nor any psychological and emotional aspects. 

## 5. Conclusions

In line with current dietary guidelines and due to the relapsing nature of CD and unpredictable flares of activity, it is not surprising that the burden of IBD is higher for CD patients, regardless of their disease activity when compared with UC and CelD patients. Although CelD involves a life-long GFD diet, symptoms from small quantities, accidental gluten ingestions, may be mild and overlooked by patients. Furthermore, the development of the gluten-free product industry has made these products extremely accessible with a considerable variability, making CelD more “manageable”. These so-called advantages of GFD upon a specific CD diet are responsible for the reduced impact on the social aspects associated with food and eating in CelD patients, like meeting with peers, overnight staying and preparing a meal.

Despite our study’s limitations, it offers several notable strengths. To the best of our knowledge, this research represents the first comparative assessment of a crucial aspect shared by two major gastrointestinal disorders. We also sought to uncover key determinants of FR-QoL and compare our findings with the existing literature. This approach allows us to both contribute new insights and validate or challenge previous results in the field. 

An extension of this research, hopefully, will improve our understanding of the impact of food and eating behaviors on the patients’ well-being which can lead to a more tailored dietary approach and better communication with the patient in order to improve compliance to such restrictive diets. Furthermore, by broadening our research in other centers, we could identify dependable risk and protective factors which could be targeted for intervention in order to enhance FR-QoL in these conditions. 

## Figures and Tables

**Table 1:** Demographics and clinical characteristics of the patients.

	Crohn’s Disease (CD) n = 17	Ulcerative Colitis (UC) n = 17	Celiac Disease (CelD) n = 17	*p*-Value
Sex [n (%)]				0.379
Male (n = 21)	9 (52.9%)	5 (29.4)	7 (41.2)	
Female (n = 30)	8 (47.1%)	12 (70.6)	10 (58.8)	
Median age at diagnosis, years [IQR]	13.2 [10.85–15]	12.3 [9.15–14.4]	4 [2–7.9]	<0.001
Median age at inclusion, years [IQR]	16 [12–16.75]	16 [14–17]	13 [10.8–16.4]	0.383
Median duration of evolution, years [IQR]	17 [9.5–45.5]	22 [9.5–60]	102 [75–132.6]	<0.001
UC extension			-	<0.001
E1	-	2 (11.8)		
E2	-	2 (11.8)		
E4	-	13 (76.4)		
CD location [n (%)]			-	0.824
L1	6 (35.3)	-		
L2	4(23.5)	-		
L3	7 (41.2)	-		
L4a	1 (5.9)	-		
CD behaviour [n (%)]			-	0.029
B1	13 (76.5)	-		
B2	4 (23.5)	-		
B3	-	-		
P	2 (11.8)	-		
PGA [n (%)]			-	0.648
0	4 (23.5)	7 (41.2)		
1	7 (41.2)	4 (23.5)		
2	5 (29.4)	4 (23.5)		
3	1 (5.9)	2 (11.8)		
Treatment regimen [n (%)]				
Diet (CDED ± PEN)	4 (23.5)	-		
5-ASA	2 (11.8)	8 (47.1)		
IMM (AZA)	6 (35.3)	3 (17.6)		
Biologics	6 (35.3)	6 (35.3)	-	0.640 *
IFX	4 (23.5)	6 (35.3)		
ADA	2 (11.8)	0 (0)		
Topic steroids/systemic steroids	1 (5.9)	1 (5.9)		
Strict GFD	-	-	16 (94.1)	
Nutritional status [n (%)]				0.908
Healthy weight	12 (70.6)	14 (82.3)	12 (70.6)	
Underweight	4 (23.5)	2 (11.8)	4 (23.5)	
Overweight	0 (0)	1 (5.9)	1 (5.9)	
Class I obesity	1 (5.9)	0 (0)		
History of surgery [n (%)]	6 (35.3)	0 (0)	-	0.018

* Computed only for biologic therapy. 5-ASA, 5-aminosalicilic acid; ADA, adalimumab; AZA, azathioprine; CD, Crohn’s disease; CDED, Crohn’s disease exclusion diet; CelD, celiac disease; GFD, gluten-free diet; IFX, infliximab; IMM, immunomodulator; IQR, interquartile range; PEN, partial enteral nutrition; PGA, physician’s global assessment; UC, ulcerative colitis.

**Table 2:** FR-QoL scores according to disease extension and behavior.

	CD n = 17	UC n = 17	*p*-Value
UC extension (n)			0.504
E1	-	60.5 ± 2.1	
E2	-	61 ± 2.8	
E4	-	73.5 ± 20	
CD location [n (%)]		-	0.418
L1	61.3 ± 3.4		
L2 + L2L4a	59 ± 4.8		
L3	69.3 ± 18.4		
CD behaviour [n (%)]		-	0.770
B1 + B1p	66 ± 18.9		
B2	63 ± 8.3		
History of surgery [n (%)]		-	0.291
positive	61.8 ± 7		
negative	70.3 ± 19		

CD, Crohn’s disease; UC, ulcerative colitis.

## Data Availability

Data are contained within the article.
